# Systematic investigation of the mechanism of herbal medicines for the treatment of prostate cancer

**DOI:** 10.18632/aging.204516

**Published:** 2023-02-10

**Authors:** Jinghui Wang, Ran Ding, Ting Ouyang, Honglei Gao, Hongxing Kan, Yan Li, Qiongying Hu, Yinfeng Yang

**Affiliations:** 1 School of Integrated Chinese and Western Medicine, Anhui University of Chinese Medicine, Hefei 230012, Anhui, China; 2 School of Medical Informatics Engineering, Anhui University of Chinese Medicine, Hefei, China; 3 Key Laboratory of Industrial Ecology and Environmental Engineering (MOE), Faculty of Chemical, Environmental and Biological Science and Technology, Dalian University of Technology, Dalian 116024, China; 4 School of Medicine, Taizhou University, Taizhou 318000, Zhejiang, China

**Keywords:** herbal medicines, prostate cancer, bioinformatics analysis, MD simulations, mechanism of action

## Abstract

Due to various unpleasant side effects and general ineffectiveness of current treatments for prostate cancer (PCa), more and more people with PCa try to look for complementary and alternative medicine such as herbal medicine. However, since herbal medicine has multi-components, multi-targets and multi-pathways features, its underlying molecular mechanism of action is not yet known and still needs to be systematically explored. Presently, a comprehensive approach consisting of bibliometric analysis, pharmacokinetic assessment, target prediction and network construction is firstly performed to obtain PCa-related herbal medicines and their corresponding candidate compounds and potential targets. Subsequently, a total of 20 overlapping genes between DEGs in PCa patients and the target genes of the PCa-related herbs, as well as five hub genes, i.e., CCNA2, CDK2, CTH, DPP4 and SRC were determined employing bioinformatics analysis. Further, the roles of these hub genes in PCa were also investigated through survival analysis and tumour immunity analysis. Moreover, to validate the reliability of the C-T interactions and to further explore the binding modes between ingredients and their targets, the molecular dynamics (MD) simulations were carried out. Finally, based on the modularization of the biological network, four signaling pathways, i.e., PI3K-Akt, MAPK, p53 and cell cycle were integrated to further analyze the therapeutic mechanism of PCa-related herbal medicine. All the results show the mechanism of action of herbal medicines on treating PCa from the molecular to systematic levels, providing a reference for the treatment of complex diseases using TCM.

## INTRODUCTION

Prostate cancer (PCa) is a widespread adenocarcinoma of the urinary system, ranking the second causes of cancer-related deaths in American males [1]. Due to the accelerated aging of the population, the incidence of PCa has risen significantly in recent years [2]. Presently, the treatment of prostate cancer mainly includes surgery, radiotherapy, cryosurgery, chemotherapy, and/or hormonal therapy [3]. However, chemotherapy and radiation therapy exhibit severe toxicity on normal tissues and hormone therapy for prostate cancer also has various unpleasant side effects, such as inducing cardiovascular diseases [3]. Therefore, more and more people seek complementary and alternative medical therapies.

Traditional herbal medicine is a whole medical system originated from several thousand years of clinical experience [4, 5]. Nowadays, increasing PCa patients worldwide are starting to use herbal medicines for treatment [6]. However, herbal medicine is a complex system and often possess dozens or even hundreds of various chemical components with diversified structures, making factually the determination of Traditional Chinese Medicine (TCM) content an indispensable while quite challenging work in TCM research. In addition, the multiple targets and multiple biological pathways of herbal medicine also makes it extremely difficult to analyze the molecular mechanism of herbal medicine. Using experimental approaches alone can hardly systematically and scientifically evaluate the function mechanism of TCMs. A computational method to solve this problem is still unavailable up to date. Owing to these facts, the development and application of computational technology is a necessity to overcome these difficulties.

Presently, to explore the mechanism of herbal medicines on treating PCa from a systematic level, a comprehensive approach combining knowledge mapping, pharmacokinetic evaluation, multi-targets fishing, bioinformatics analysis and computer experiments validation was proposed. Firstly, using the bibliometric analysis and text statistics mining, 10 most frequently used herbs of PCa and their corresponding constituents were obtained. Secondly, an *in silico* pharmacokinetic model system was employed to screen out the potential active compounds of anti-PCa herbs and then the targets of these potential active ingredients were fished. Thirdly, the overlapping genes among differentially expressed genes (DEGs) in PCa patients and the target genes of the PCa-related herbal medicines were obtained. Subsequently, five hub genes were applied to perform survival and tumour immunity analysis, revealing their critical roles in PCa.

**Figure 1:**
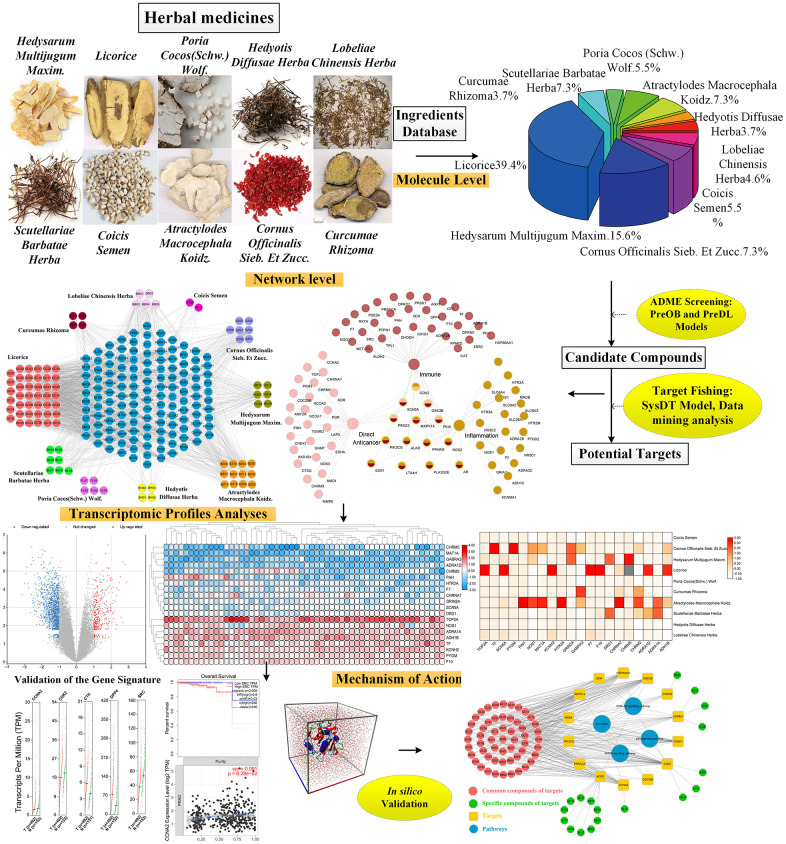
The detailed workflow of this study.

Additionally, computer experiments validation including the simulations of molecular dynamics (MD) and molecular docking were carried out to verify the reliability of interactions between the key drugs and the hub target genes. In final, through integrated analysis, four signaling pathways including PI3K-Akt, MAPK, p53 and cell cycle pathways were determined, demonstrating that herbal medicines show the therapeutic effects on treating PCa by acting on the target genes in these pathways. Figure 1 shows the framework of this study.

## MATERIALS AND METHODS

### Data sources

To extract the relevant herbal medicine for prostate cancer, Web of Science (WoS), a crucial global database containing large amounts of academic literatures, was employed to perform bibliometric analysis. Using the keywords “prostate cancer” and “herbal medicine”, the related literatures were collected in WoS from January 2001 to December 2021. Presently, VOSviewer was used as the bibliometric analysis tool to extract the hot herbal medicine against PCa.

### Extracting PCa-related herbs

Further, to obtain the herbal medicines associated with PCa, a data mining approach based on the keywords “prostate cancer” and “herbal medicine” was carried out from TCMSP (https://old.tcmsp-e.com/tcmsp.php), PubMed and the clinical trial database (https://www.clinicaltrials.gov). Then, a statistical index (number of articles on herbal medicine for PCa / number of articles on herbal medicine) as a ratio was used to assess the relationship between herbal medicine and PCa. Herein, P value is used to evaluate the relationship between herbs reported in literature and PCa and when P-value < 0.01, this herb is considered to have strong association with PCa [7].

$$P=1-\sum_{i=1}^{k-1}f(i)=1-\sum_{i=0}^{k-1}\frac{\left(\begin{array}{c} K\\ i \end{array}\right)\left(\begin{array}{c} N-K\\ n-i \end{array}\right)}{\left(\begin{array}{c} N\\ n \end{array}\right)}$$ (1)

where *N* denotes the total number of articles retrieved from the databases, *K* represents the number of papers related to PCa, *n* is the number of papers related to a herbal medicine and *k* shows the number of papers related to prostate treatment cancer by the corresponding herb, respectively.

### Database construction of chemicals in herbs related to PCa

All ingredients in the herb medicines associated with PCa are obtained through large-scale excavation of databases of TCM and literature Mining. For the collected glycosides, considering that these compounds will be hydrolyzed into glycosides by intestinal flora when they pass through the human small intestine [8], the corresponding glycosides are also added to the component database. The structures of these compounds are downloaded through the Chemical Book and NCBI PubChem websites, and further optimized by performing the standard Tripos force field in Sybyl 6.9 (Tripos Associates, St. Louis, MO).

### Screening of active components in PCa-related herbs

The pharmacokinetic properties of the collected compounds were assessed by two computationally models, i.e., PreOB (predict oral bioavailability) and PreDL (predict drug-likeness).

### PreOB

For the potential compounds, OB, an essential pharmacokinetic parameter, is a good indicator to reflect the efficiency of oral drugs entering the systemic circulation [9]. Herein, a reliable *in silico* model PreOB [10] was employed to predict the ingredients of the herb medicines associated with PCa. This model is developed based on a support vector regression equation with an insensitive loss function and an optimization constant, as depicted in formula (2).

$$f(x)=\sum_{i=1}^{n}\left(\alpha_{i}-\alpha_{i}^{*})K(x,x_{i}\right)+b$$ (2)

Where α_i_ and α_i_^*^ are the Lagrange multipliers, b is the regression parameter K and (x, x_i_) is the kernel function, which uses the radial basis function, as shown in formula (3).

$$K(x,\;x_{i})=\exp(-\frac{{\left\|x-x_{i}\right\|}^{2}}{\gamma^{2}})$$ (3)

Where γ is the nuclear parameter.

### PreDL

Drug-likeness (DL) represents the similarity of a compound’s structure to that of a known drug [11]. A compound with good DL is not necessarily a drug, but has the potential to be a drug. Such a compound is called drug-like molecule or drug analog molecule. Currently, PreDL model was carried out to compute the DL index based on the following formula:

$$\text{F}(\text{x},\;\text{y})=\frac{\text{x}\cdot \text{y}}{{\left|\text{x}\right|}^{2}+{\left|\text{y}\right|}^{2}-\text{x}\cdot \text{y}}$$ (4)

Where x is the descriptors of the herbal ingredients associated with PCa, and y denotes the mean of the total molecular descriptors of all drugs in the Drugbank database.

To sum up, the screening thresholds for the two models are set as OB ≥ 30% and DL ≥ 0.18, respectively. The compounds in herbal medicines can be identified as potential active ingredients if they meet these two screening conditions at the same time.

### Drug targeting for PCa-related herbs

Generally, drug molecules can exert their biological effects only by interacting with their targets. Therefore, in order to accurately and comprehensively predict the targets of candidate compounds in herbal medicines associated with PCa, SysDT model [12], which is developed on the basis of random forest method (RF) and support vector machine method (SVM) was employed. In this model, the compound-target interaction satisfying RF ≥ 0.8 and SVM ≥ 0.7 is considered effective and this target protein is regarded as a potential target of the active ingredient.

In final, the obtained target proteins are further subjected to TTD database to fish the PCa-related targets.

### Construction and analysis of the biological network

Biological network can provide an intuitive display of the interaction between nodes. Presently, three nets, i.e., compound-target (C-T), target-function (T-F) and compound-target-pathway (C-T-P) networks are established and analyzed by using Cytoscape 3.3.8 [13]. In these networks, compounds, targets, protein function or signaling pathways are represented by Nodes, while compounds-targets, targets-functions, or targets-pathways are represented by Edges. Two important topological parameters ‘degree’ and ‘betweenness’ in the network are calculated by the Network Analyzer and CentiScaPe 1.2 plugged-in the Cytoscape.

### Identification of differentially expressed genes in PCa patients

To identify the differentially expressed genes (DEGs) in the prostate tumor, gene expression profile of GSE134073 was downloaded from Gene Expression Omnibus (GEO) database [14]. This affymetrix macroarray data contains 40 tissue specimen from the patients with PCa and 8 human benign prostate hyperplasia (BPH) tissue samples. Employing the Limma package in R version 4.2.0, the DEGs were analyzed and the cut-off criterion for screening DEGs is set as the false discovery rate (FDR) < 0.05 and |log2 fold change (FC)| > 1.

### Biological function and pathway enrichment analysis

To reveal significantly enriched biological processes and molecular function of the obtained genes, Gene Ontology (GO) analysis was carried out through the R package clusterprofiler [15]. Only the GO functional terms with P-value < 0.05 was selected. Additionally, KEGG (Kyoto Encyclopedia of Genes and Genomes) pathway enrichment analysis was also performed to investigate the systemic effects of compounds in herbal medicine on treating diseases. Using the package ggplot2, GO terms and KEGG pathways were visualized and an incorporated PCa-related pathway was also assembled.

### Screening PCa-related hub genes and survival analysis

To filter the hub genes in human PCa tumors, the Gene Expression Profiling Interactive Analysis (GEPIA) (http://gepia.cancer-pku.cn/) was applied based on TCGA samples. Hub genes were subjected to survival analysis in GEPIA and Genes with |log2(FC) | > 1 and P < 0.01 were considered significant. In addition, differential analysis for these hub genes was carried out by using the Human Protein Atlas online tool.

### Correlation between hub genes expression and immune cell

In order to explore the correlation between hub gene expression and immune cells, TIMER (https://cistrome.shinyapps.io/timer/), a website dedicated to analyzing tumor immune correlation, was employed. Through TIMER, the mRNA expression data for the obtained hub genes in TCGA PCa tumor samples and their correlations with tumor infiltration of 6 immune cell types including B cells, CD4+T cells, CD8+ T cells, neutrophils, macrophages, and dendritic cells was analysed.

### MD simulations

Further, for exploring the mechanism of action and the binding modes of these key genes and their key compounds, we selected four C-T interactions for MD simulations. The X-ray crystal structures of these targets were obtained from RCSB PDB database. Using the GROMACS software [16] with GROMOS96 force field [17]. The cutoff distances of Coulomb and van der Waals interactions are calculated to be 1.0 and 1.4 nm, respectively. Before simulation, the steepest descent integrator is used to minimize the energy of the unconstrained full system. Additionally, using the steepest descent integrator, energy minimizations were performed for the systems without constraints and the systems were equilibrated through 500 ps MD simulations at 300 K [18]. Employing the particle-mesh-Ewald (PME) method [19], the long-range electrostatics was calculated. In order to ensure the stability of the entire system, 100 ns with a 2 fs time step MD simulations of protein-ligand complexes were performed.

### The binding free energy

By using the g_mmpbsa algorithm, the binding free energy for four drug-target complexes were calculated based on the MM-PBSA [20]. The difference in energy of the drugs, targets and drug-target complexes from drug-target binding affinities were computed by the followed equations:

$$\text{Δ}G_{\text{bin}}{}_{\text{d}}=\text{Δ}E_{\text{MM}}+\text{Δ}G_{\text{sol}}-T\text{Δ}S$$ (3)

$$\text{Δ}E_{\text{MM}}=\text{Δ}E_{\text{internal}}+\text{Δ}E_{\text{electrostatic}}+\text{Δ}E_{\text{vdw}}$$ (4)

$$\text{Δ}G_{\text{sol}}=\text{Δ}G_{\text{PB}}+\text{Δ}G_{\text{SA}}$$ (5)

where Δ*E*_MM_ represents the relation energy among the drug and the protein. Δ*G*_sol_ is the total energy of polar contribution and non-polar contribution of Δ*G*_PB_ (electrostatic solvent free energy) and Δ*G*_SA_ (non-electrostatic solvent component), respectively.

### Bioactivity of *K*_i_

The inhibitory constant *K*_i_ was calculated from the MM-PBSA. The equation of *K*_i_ is listed as follow [21, 22]:

$$K_{\text{i}}=e^{\frac{-\text{Δ}G_{\text{bind}}}{RT}}$$ (6)

Where R and T (298.15 K) represent gas constant (1.987×10^–3^ kcal/K-mol) and absolute temperature of drug-target complex, respectively. Δ*G*_bind_ denotes the binding free energy.

### Statistical analysis

All the data are presented as mean± S.E.M. Employing Prism software (GraphPad, CA, USA), the statistical analysis was carried out. Additionally, t-test was used to assess the statistical significance. When P value is less than 0.05, the result is considered statistically significant.

## RESULTS

### ADME screening

**Figure 2:**
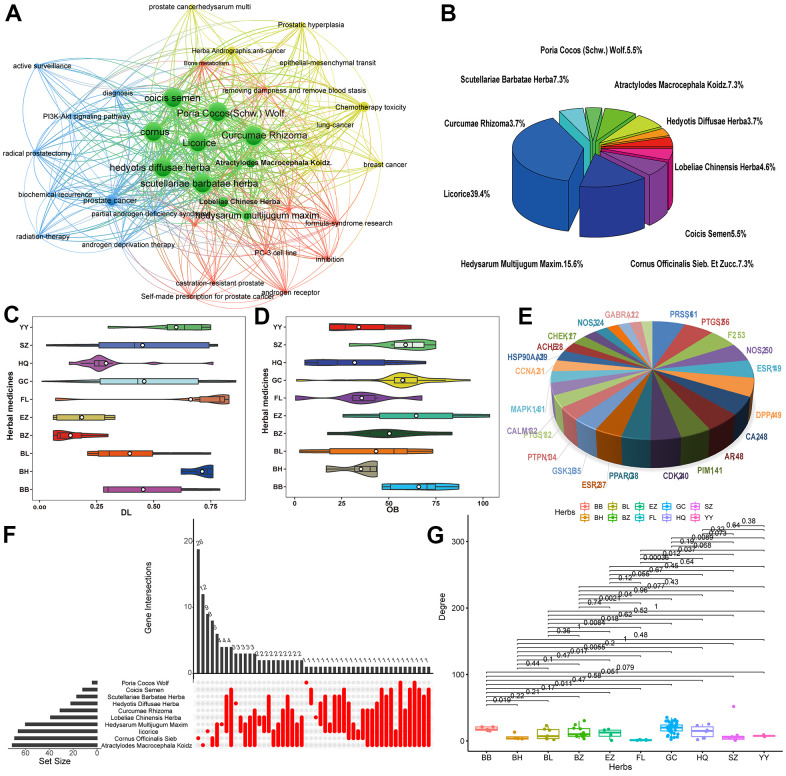
(**A**) Network map showing keywords in PCa-related literature. (**B**) Pie chart of the proportion of TCM targets in the treatment of PCa. (**C**, **D**) Violin diagram of OB and DL content. (**E**) Key target gene in herbs. (**F**) Correlation map of herbs target genes. (**G**) Degree value of target genes in the treatment of prostate cancer with TCM.

According to bibliometric analysis, text statistics mining and much experiences based on the occurrence in prescription, ten herbs i.e., *Hedysarum Multijugum Maxim., Licorice, Poria Cocos (Schw.) Wolf.*, *Hedyotis Diffusae Herba*, *Lobeliae Chinensis Herba, Scutellariae Barbatae Herba, Coicis Semen, Atractylodes Macrocephala Koidz., Cornus Officinalis Sieb. Et Zucc.* and *Curcumae Rhizoma*, which are significantly correlated with PCa, were obtained (Figure 2A). Figure 2B depicts the proportion of the active ingredient of each herb, in which *Licorice* accounts for the highest proportion, 39.4 %. Using the PreOB prescreening model, the OB values of the ingredients in these herbs are calculated and totally 87 compounds satisfying the query requirements (OB ≥ 30%), accounting for a large proportion in all herbs (Figure 2D). In fact, many compounds with high OB values have already been identified as bioactive ingredients. For example, scoparone (OB = 75%) is isolated from *Lobeliae Chinensis Herba* and has been widely used in TCM. Through inhibiting the STAT3 activity, scoparone exhibited a certain anti-tumor effect on human PCa DU145 cells and also contains the biological activities of vasorelaxant, anti-coagulant, anti-inflammatory, hypolipidemic anti-oxidant and effects [23].

Besides, employing the PreDL model, a total of 97 candidate compounds with satisfied DL values were obtained from the 10 herbs and the proportion of DL is depicted in Figure 2C. Among them, many of them have important anticancer activities reported in the literature. For instance, β-sitosterol (DL = 0.71), the most common phytosterol, reduces the level of PSA released in the medium, showing an inhibitory effect on tumor growth [24]. Actually, studies have found that β-sitosterol can induce apoptosis in cultured LNCaP PCa and MDA-MB-231 breast cancer cell lines [25]. Moreover, Didemethoxycurcumin, as an active compound reported in various types of cancers, can significantly inhibit proliferation, migration and invasion of cultured PC-3 cells, showing the potential for treating PCa [26].

After screening with both models simultaneously, a total of 75 chemicals meeting the screening criterions of OB ≥ 30% and DL ≥ 0.18 were obtained, showing their good pharmacokinetic properties. In addition, some compounds with relatively low OB or DL values, but they were also added into the active chemical database for further because the biological activities of these compounds have been confirmed. Taking scutellarin of *Scutellariae Barbatae Herba* for an example, with a low OB value of 2.64%, it has been reported to promote the cobalt chloride-mediated apoptosis in PC12 cells, a kind of human PCa cell line [27]. Through increasing the Bcl-XL expression and inhibiting the activity of caspase-3, scutellarin prominently decreased the percentage of apoptosis population, p38 MAPK phosphorylation and ROS production in CoCl2-treated PC12 cells, presenting its protective effects [28]. Furthermore, isoliquiritigenin with the lowest DL of 0.01, showed the anti-oxidative and anti-tumour activities and considered as a potent antimetastatic agent. Previous study showed that isoliquiritigenin can significantly prevent the metastasis and invasion of cancer cells by inducing apoptosis of PCa cells [29].

In final, 109 potential ingredients (Supplementary Table 1) are totally collected from 10 anti-PCa herbs in the present work.

### Target identification

Using the SysDT model [7], a total of 139 potential targets were identified for 109 candidate compounds, generating 1366 ligand-target interactions. Figure 2E depicts the key target gene in anti-PCa herbs obtained above and the distribution of gene targets in various herbs was shown in Figure 2F. The results present that the majority of active chemicals hit more than one target protein and most targets are linked with different numbers of compounds, showing the promiscuous actions of herb ingredients and the multicomponent characteristics of herbs (Figure 2G). For instance, tert-butanol has the highest number of degree, followed by shinpterocarpin (degree = 35) and glyasperins M having 32 target proteins. Also, the chemical luteolin acts not only as an inhibitor of XDH and IL4, but also as an antagonist of PPARFγ [30, 31]. Moreover, 18 targets (22.5%) are commonly modulated 23 drugs, implying the synergism or cumulative effects of these drug molecules.

### Network construction and analysis

Generally, herbal drugs exert therapeutic effects through acting on their corresponding protein targets, and drug responses, including therapeutic effects and side effects, are affected by the topological properties of the networks. Analyzing the networks at systems level can provide useful information for exploring the different types of molecular relationships. Presently, we employed the network pharmacology to explore how a multi-component treatment system like the ten herbs as mentioned above exert their therapeutic effects on the treatment of PCa.

**Figure 3:**
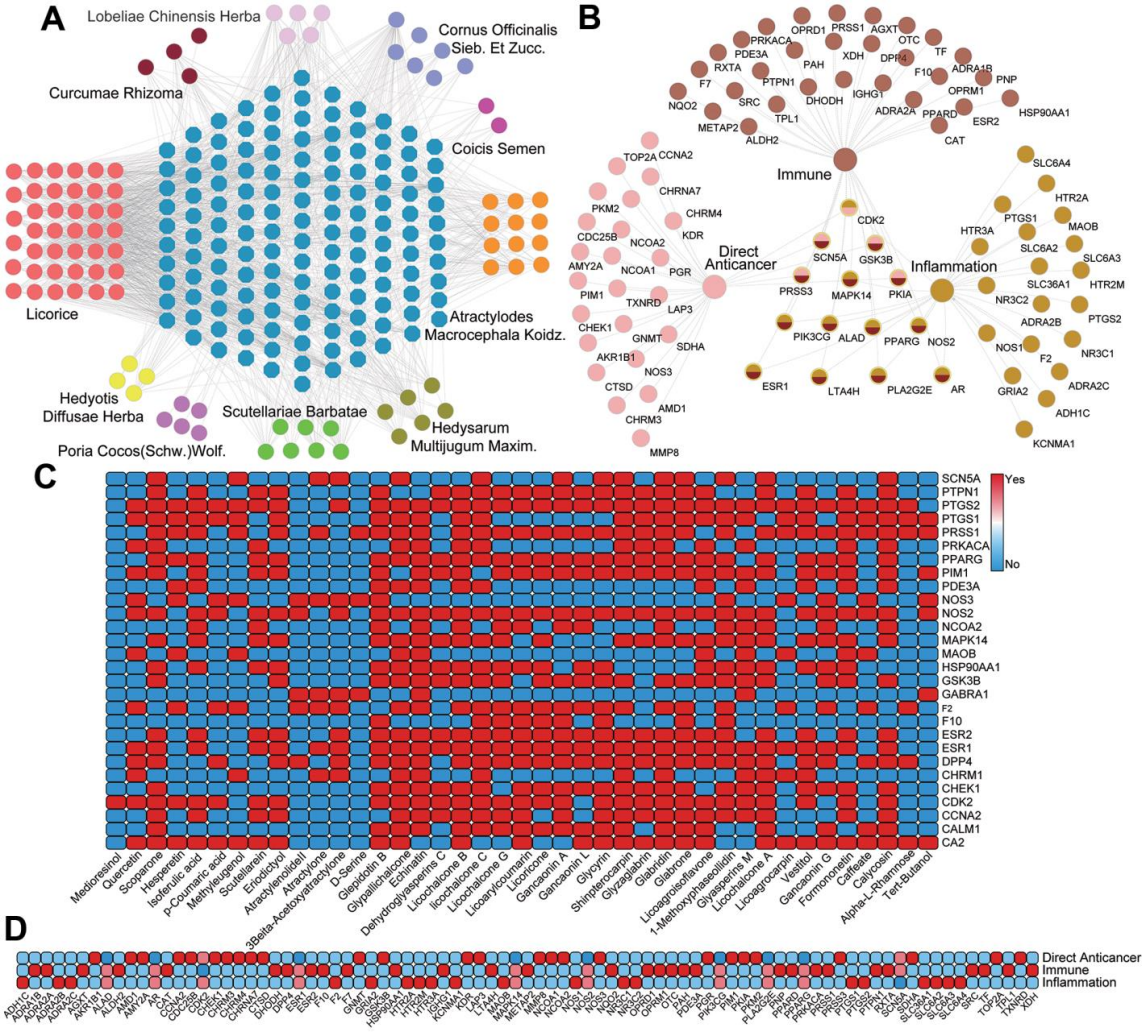
(**A**) The C-T network. Circles and octagons represent the chemicals from ten herbals and all their potential targets, respectively. (**B**) T-F network. 28 targets related to direct anticancer therapy 39 targets related to immunization therapy and 28 targets related to inflammation therapy. The overlapped targets in the middle are the common targets of all three therapies. (**C**) The heatmap of C-T interaction analysis. (**D**) The heatmap of T-F network analysis.

As is shown in Figure 3A, using 109 active ingredients of 10 herbs and their corresponding 139 target proteins, the C-T network was established. In this net, the active compounds and their targets are represented by circles and octagons, respectively. The interactions between the compounds and the targets were shown in Figure 3C, indicating that a total of 109 genes are identified as the targets corresponding to the active ingredients. Moreover, a crucial quantitative parameter of C-T network, the degree has been explored, which refers to the number of edges of a node. Through analysis of the net, we found that among the active chemicals, Tert-Butanol (SZ04) exhibits the largest number of degree (Degree = 52), followed by Shinpterocarpin (GC20) with 35 targets, and Glyasperins M (GC37) with 32 targets, showing the multi-target feature of compounds in TCM. Since the primary therapies of PCa are the direct anti-cancer treatment, immunotherapy and anti-inflammation strategy, presently, the targets which are associated with these three therapies have been carefully selected for the study of synergistic effects for different components of TCM. Subsequently, the T-F network employing the targets with their functions was also constructed (Figure 3B) and the heatmap for the detailed targets and their corresponding functional classifications is described in Figure 3D.

Further, analysis of the C-T function network (Figure 3B) reveals that there are 28 targets related to direct anti-cancer therapy, among which 5 targets associated with two functions. For instance, the target protein Androgen Receptor (AR) has a relatively strong interaction with 62 active molecules, implying the multi-component characteristics of herbs and indicating the significant effects of PCa-herbs in the directly controlling prostate carcinogenesis. Indeed, the widespread expression of AR in tumors is key to assess the PCa progression and treatment outcomes, and the majority of PCa tumors (80-90%) rely on androgen for their growth and survival [32, 33]. For these reasons, endocrine therapy of PCa is directly employed to reduce the serum androgens and inhibit the activity of AR [34, 35]. Additionally, 90% of cancer mortality is related to metastasis, which is the most important component in cancer pathogenesis [36]. Currently, there are many targets regulating the metastatic tumor in the C-T function network (Figure 3B), among which ADRB2 (Beta-2 adrenergic receptor, Degree=30) and SCN5A (Sodium channel protein type 5 subunit alpha, Degree=26) show the significant effects in the systematic controlling the oncogenic cells migration. In this C-T function network, 29 compounds bind to ADRB2 and 25 components bind to SCN5A, respectively, indicating that the characteristics of multiple components and multiple targets in TCM.

In addition, we found a total of 39 potential target proteins that are significantly related to immunity, which includes ESR1, F10, PRKACA, RXRA and PDE3A, etc. (Figure 3B). Among them, the degree of ESR1 is the highest (Degree = 62), implying its crucial role in regulating immune response. Actually, receptors for estrogens (ERs) regulate the innate and adaptive immune system cells, and the development of immune cells. It is reported that ER activities can enhance and inhibit the innate immune responses of macrophages and dendritic cells [37]. Meanwhile, it can help to attain the immune system balance by modulate chronic inflammatory disorder. Besides, DPP4, a ubiquitously expressed transmembrane protein, is connected with 14 compounds. Recent studies have suggested that DPP4 can regulate the tumor growth [38]. *In vivo* post-translational modification of chemokines by DPP4 exerts an inhibition on the migration of T cell to tumors and DPP4 inhibition might therefore be a useful adjuvant treatment for immunotherapy in cancer patients [39].

Besides, prostatic inflammation is the major predisposing factor for prostatic cancer [34]. Chronic prostate inflammation may initiate and accelerate PCa progression. By further observing the C-T network, we found that many targets with large number of degree are associated with inflammatory mediators, such as PTGS2, NOS2, F2, PPARG and PTGS1. Among these targets, PTGS1, a key enzyme in the synthesis of prostaglandin connected by 41 compounds, is involved in maintaining tissue homeostasis and cellular signaling, while PTGS2 with the highest number of compound-target interactions (Degree = 68), affects PCa progression and development, mainly regulated by neovascularization and increased resistance to apoptosis [40–42]. Emerging genetic and clinical studies have suggested that in animal models, increased expression of PTGS2 induces tumorigenesis, while inhibition of PTGS2 results in decreased tumor incidence and progression [43–47]. In addition, recent studies also suggest that PPARG has a regulatory role in the control of inflammatory responses, showing the potential therapeutic applications of PPARG in inflammation-related PCa [48].

### Identification of PCa-related DEGs

**Figure 4:**
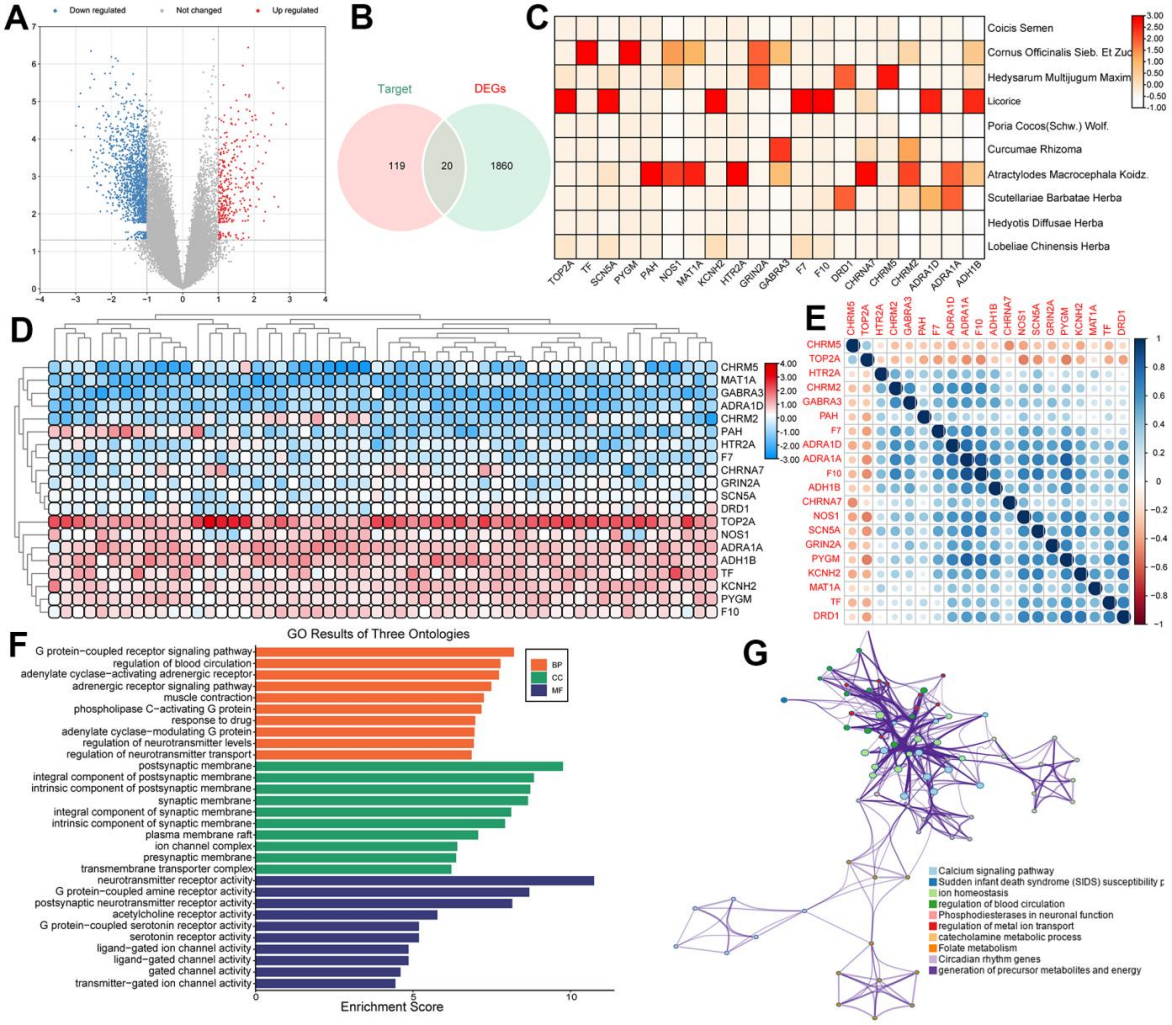
(**A**) The volcano plots for the 1880 DEGs in the GSE134073 dataset. (**B**) The overlapped genes between DEGs in PCa and the target genes of the PCa-related herbs. (**C**) The correlation between overlapped genes and drugs. (**D**) Heatmaps of significantly differentially expressed genes based on high Gleason score and low Gleason score groups. (**E**) A heatmap of the correlation of overlapped genes. (**F**) Gene Ontology (GO) enrichment of overlapped genes. (**G**) Visualized functional enrichment and gene interaction analysis results.

In order to find the intersection of DEGs in PCa and the target genes of the PCa-related herbs, we firstly identified 1880 DEGs in GSE134073 (363 upregulated and 1517 downregulated) (Figure 4A). Then, a total of 20 overlapped genes between DEGs in the prostate tumor and the target genes of the PCa-related herbs were obtained by using Venn diagrams (Figure 4B). The correlation heatmap of the 20 overlapped target genes and the herbal drug associations is shown in Figure 4C, demonstrating that the effects of drugs on tumor tissues with different Gleason scores are closely related. Additionally, the expression levels of the overlapped genes between high and low Gleason score groups are depicted in Figure 4D, 4E illustrates the heatmap of the correlation of overlapping genes. Besides, Figure 4F, 4G show the Gene Ontology (GO) functional enrichment of the overlapped 20 target genes, which reveals that these genes participate in the main biological processes associated with PCa. For example, the neurotransmitter receptor-mediated signaling pathway can be used as a regulator of carcinogenesis [49]. There is increasing evidence that cancer cells can use neurotransmitter-triggered signaling pathways to activate uncontrolled proliferation and spread. In addition, neurotransmitters can affect immune cells and endothelial cells in the tumor microenvironment and promote the progression of tumor including PCa [50].

### Expression and survival analysis of hub genes in PCa and normal tissues

**Figure 5:**
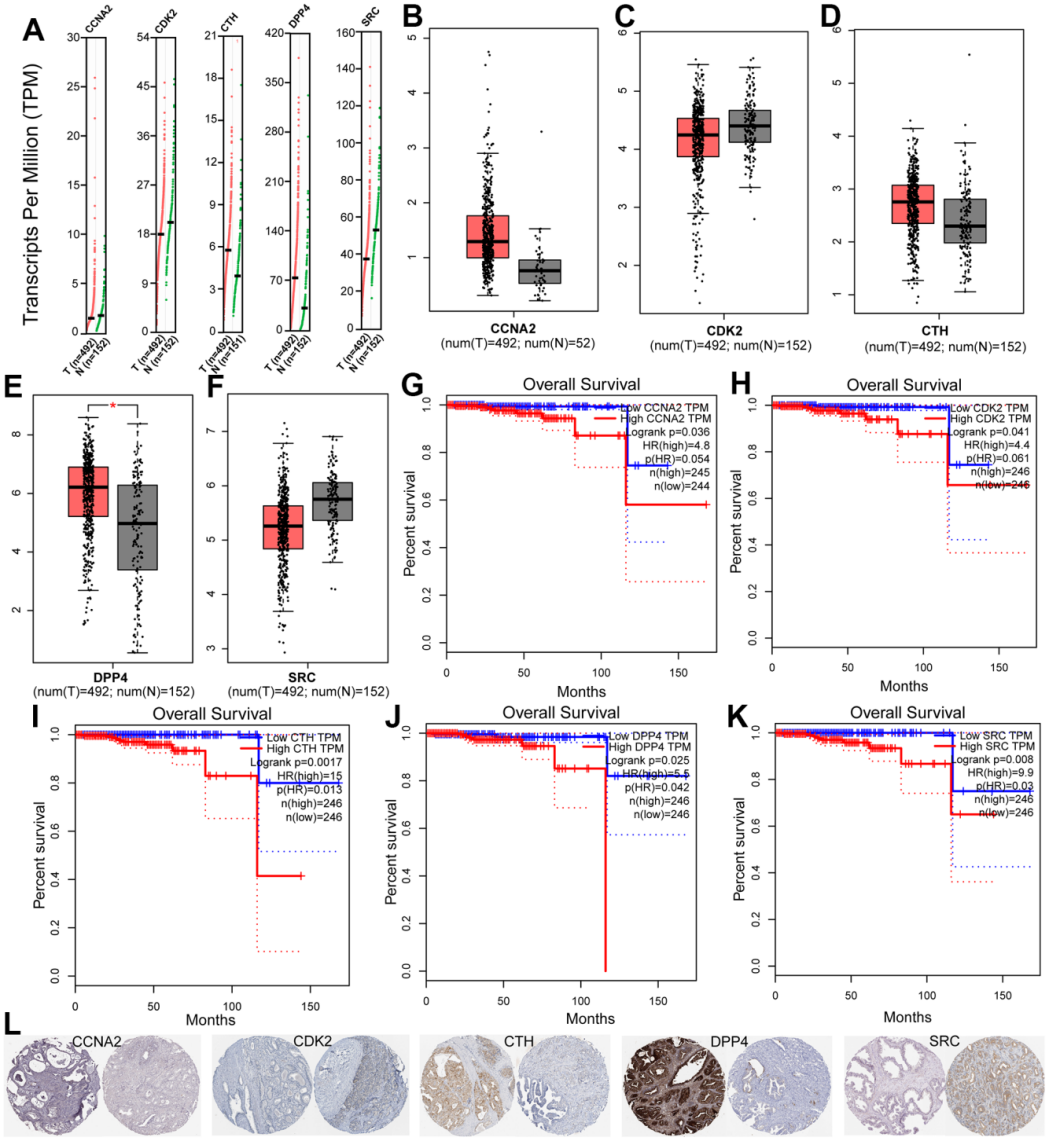
(**A–F**) The expression of five hub genes in TCGA database between PCa and peritumoral tissues. (**G**–**K**) Kaplan-Meier survival analysis of five prognostic genes in TCGA cohort. (**L**) Immunohistochemical analysis of five genes in HPA database.

To filter the hub target genes that are associated with human PCa prognosis, the GEPIA online tools was employed. As a result, five hub genes including CCNA2, CDK2, CTH, DPP4 and SRC were obtained, which are used for further analysis. The expression analysis of these hub genes in PCa and normal tissues reveals that CCNA2, CDK2, CTH and DPP4 are unregulated in PCa tissue compared with normal tissue, while SRC is down-regulated in PCa tissue compared with normal tissue (Figure 5A–5F). In addition, to explore the association between hub genes and the survival in PCa patients, the Kaplan-Meier survival analysis was employed. It was demonstrated that the cases with high expression of key genes in the TCGA cohort exhibited a poorer survival rate compared with cases without alterations (Figure 5G–5K). Besides, immunohistochemical (IHC) staining results obtained from HPA database also showed the expression status of core genes and patient profiles, which is consistent with our previous results (Figure 5L).

### Correlation between Hub gene expression and immune cell infiltration

**Figure 6:**
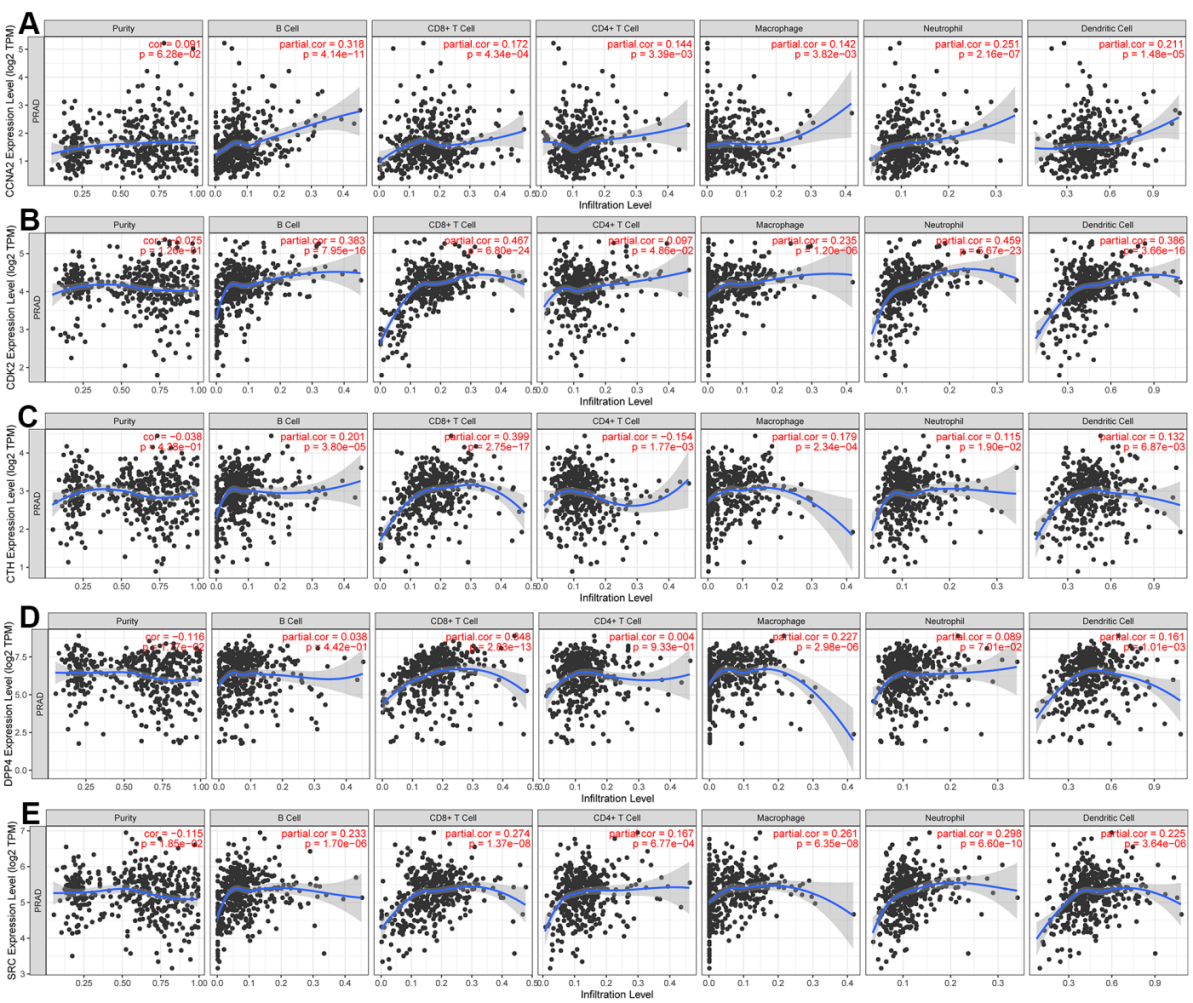
**Using the TIMER dataset to analyze the relationship between the expression of five hub genes and tumor purity and immune cells.** (**A**) CCNA2, (**B**) CDK2, (**C**) CTH, (**D**) DPP4, (**E**) SRC.

To investigate the relationship between CCNA2, CDK2, CTH, DPP4, SRC and tumor purity as well as immunity, TIMER database was used to analyze the relationship between these hub genes and immune cell infiltration in PCa. As shown in Figure 6, five Hub genes were negatively correlated with the purity of PCa. In terms of correlation with immune cells, CCNA2 and CDK2 were positively correlated with B cells, CD4+ T cells, CD8+ T cells, neutrophils, dendritic cells and macrophages. CTH, DPP4 and SRC were negatively correlated with B cells, CD8 + T cells, neutrophils and macrophages.

### Computer experiments validation of the C-T interactions

### Molecular docking for target validation

**Figure 7:**
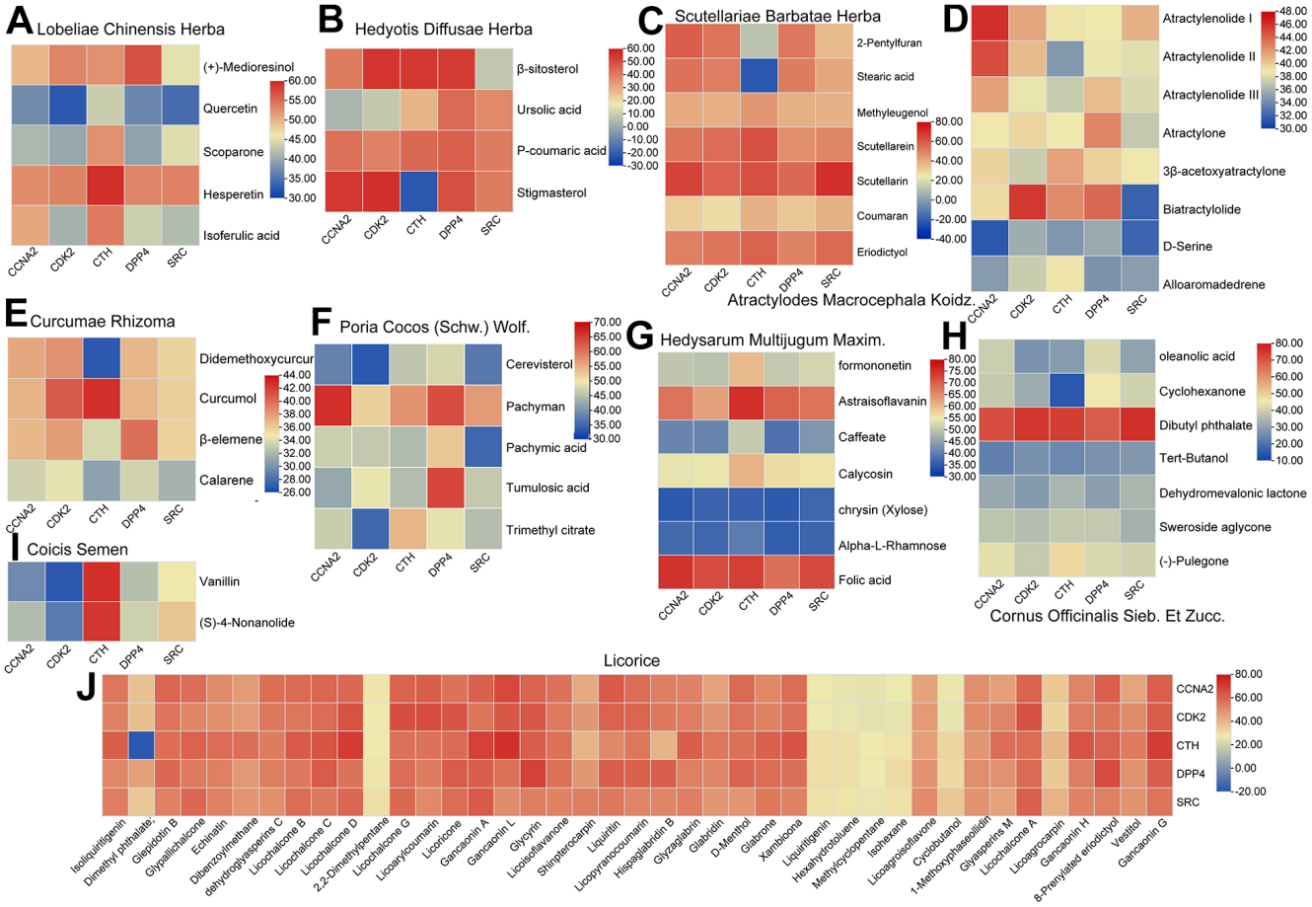
**The heat map of the docking results between the active compounds of ten herbs and five hub targets.** (**A**) Lobeliae Chinensis Herba. (**B**) Hedyotis Diffusae Herba. (**C**) Scutellariae Barbatae Herba. (**D**) Atractylodes Macrocephala Koidz. (**E**) Curcumae Rhizoma. (**F**) Poria Cocos (Schw.)Wolf. (**G**) Hedysarum Multijugum Maxim. (**H**) Cornus Officinalis Sieb. Et Zucc. (**I**) Coicis Semen (**J**) Licorice.

Molecular docking is a key method for dealing with C-T interactions, which generates posture and dimensionless fitness scores in structural molecular biology and computer-aided drug design. In the present study, in order to verify the C-T interaction between all the active ingredients of PCa-related herbs and their corresponding hub targets, the advanced molecular docking was carried out to generate the set of docking conformations. The active components from the ten herbs were docked into the five hub targets with the default settings through the Lamarckian genetic algorithm program GOLD 5.1 Score fitness function to its prediction target [51]. The heat map of the docking results was visualized through TBtools [52]. The higher score the docking results obtain, the stronger binding force these molecules and their targets are considered to possess. As shown in Figure 7, the high score of these active compounds indicates that the ligand binds well with their hub targets, which may indicate that the core active compounds have good binding activities with PCa receptor.

### The molecular dynamics simulations

To verify the reliability of the C-T interactions and to further clarify the possible binding modes and binding stability of active compound at the protein binding site, we selected two proteins from the five hub genes following the rules of randomness and performed 100 ns MD simulations for the ligand-protein complex.

**Figure 8:**
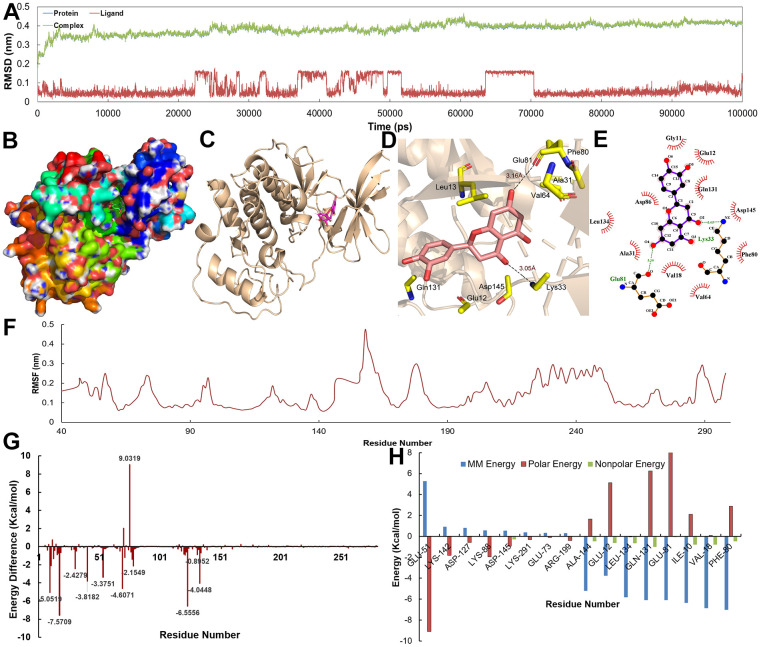
(**A**) The RMSD for CDK2 with Eriodictyol over 10 ns. (**B**–**E**) The binding mode of the CDK2-Eriodictyol complex. (**F**) The RMSF for CDK2-Eriodictyol complex. (**G**, **H**) The energy of the key amino acids from CDK2-Eriodictyol complex.

**Figure 9:**
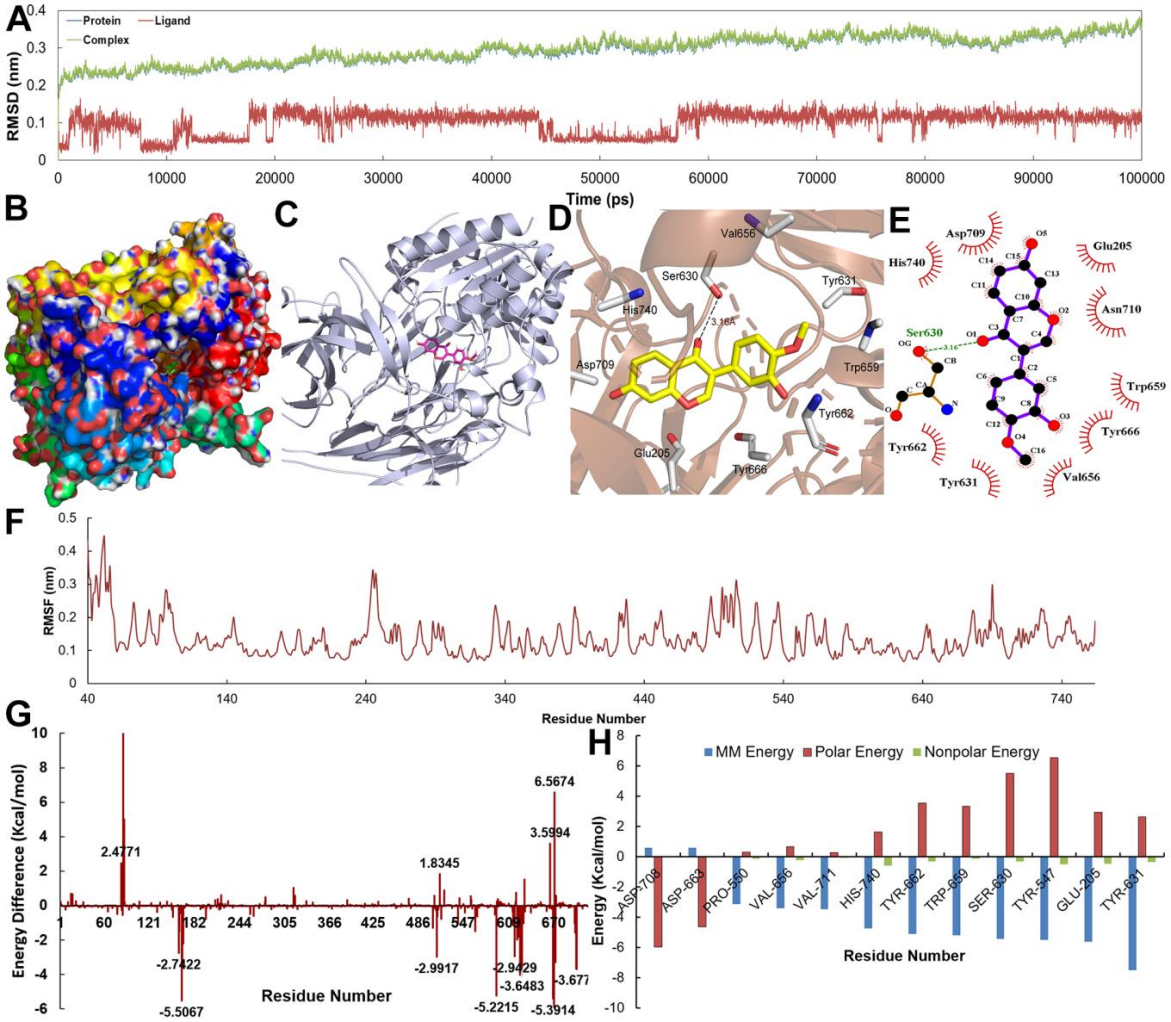
(**A**) The RMSD for DPP4 with Calycosin over 10 ns. (**B**–**E**) The binding mode of the DPP4-Calycosin complex. (**F**) The RMSF for DPP4-Calycosin complex. (**G**, **H**) The energy of the key amino acids from DPP4-Calycosin complex.

The Root Mean Square Deviation (RMSD) for the proteins with their ligands is depicted in Figures 8A, 9A. Obviously, the RMSD values of complexes, protein and ligands reached stability at approximately 3 Å after 10 ns. With regard to individual ligands, the RMSDs of Eriodictyol and Calycosin were 1.0 and 1.5 Å, respectively. The trajectory information indicates that C-T interaction tended to be stable after about 10 ns, and the binding sites of target could accommodate the corresponding active compounds without large conformational adjustment.

Figures 8B–8E, 9B–9E show the binding modes of compounds Eriodictyol and Calycosin with CDK2 and DPP4 receptor when the MD trajectories achieved equilibrium. Figure 8D, 8E depict the detail C-T interaction in the binding site. Clearly, Eriodictyol extends deeply into the binding site of CDK2, which consists of the residues Gln131, Glu12, Asp145, Lys33, Val64, Ala31, Phe80 and Leu13 via the H-bonds and hydrophobic interactions. Figure 9D, 9E indicate that Calycosin is directed interacts with a deep active site, establishing hydrogen bonding interactions with residue Ser630.

Additionally, in order to demonstrate the flexibility of binding residues in the target protein, we also calculate the root mean square fluctuation (RMSF) of each residue. Obviously, some important residues in the hydrophobic region have the lowest RMSF values, indicating that the rearrangement, variation, deviation and internal movement of the structure reduce the activity complexity of these residues and the flexibility of the binding site (Figures 8F, 9F). Furthermore, to obtain more detailed interactions between ligands and residues, the binding free energy is decomposed into a single residue in binding mode from the target protein (Figures 8G, 8H, 9G, 9H). It can be clearly seen that the main energy contributions of each key amino acid were van der Waals and nonpolar solvent free energy.

### The binding free energy analysis

In order to further validate the activities of ingredients, the binding free energy between the active ingredients and their predicted targets from the C-T interactions is performed by using MM-PBSA method. It can be clearly observed that the two active compounds show low binding free energy of -153.058 and -161.812 KJ/mol and low *K*_i_ by hitting the corresponding target (Table 1), indicating the high binding affinity to their target from the C-T network. In addition, VDW and electrostatic term play a major role in the combination, while polar solvation term is the disadvantage of these two compounds. Therefore, the beneficial interactions in the obtained complexes cannot completely compensate for the adverse effects.

**Table 1:** The detail of the energy (KJ/mol) and *K*_i_.

**Complexes**	**Δ*E*_vdwz_**	**Δ*E*_electrostatic_**	**Δ*G*_PB/GB_**	**Δ*G*_SA_**	**Δ*G*_bind_**	***K*_i_ (μM)**
CDK2-Eriodictyol	-153.058	-46.368	127.261	-16.802	-88.840	3.82E-09
DPP4-Calycosin	-161.812	-39.238	113.552	-16.058	-103.521	8.72E-32

As a result, a conclusion can be drawn that van der Waals force and nonpolar solvation energy mainly contribute to the free energy of binding.

Taken together, MD simulation provides indirect information about the biological efficacy of active compounds, which may be achieved through corresponding targets for cancer treatment, thus further validating our C-T network.

## DISCUSSION

As the most common reproductive system cancer in men, the incidence of prostate cancer is higher in Europe and the United States than that in Asia. However, the treatment of PCa has so far remained unsatisfactory, and more and more people use herbal medicines to treat PCa. Presently, a comprehensive approach combining knowledge mapping, pharmacokinetic evaluation, multi-targets fishing, network analysis and validation was proposed to explore the therapeutic mechanism of PCa-related herbal medicines.

As a result, a total of 109 potential ingredients (Supplementary Table 1) and 139 corresponding target proteins are collected from 10 anti-PCa herbs in the current work. Interestingly, GO enrichment analysis shows that the biological processes of these targets involved in are mainly grouped into the inflammation response, modulating the immune system and direct anti-cancer effects (Figure 3B), which is supported by the previous studies. For example, NR3C1, a receptor for glucocorticoids, has been widely used in the treatment of PCa patients due to its potent pro-apoptotic properties [32]. Moreover, Glucocorticoids increase androgen-independent growth of PCa cells after mutation of the glucocorticoid receptor, indicating its great significance in the development of new therapeutic modalities for treating PCa [6]. In addition, inflammatory mediators and cellular effectors are important components of the tumor local environment, especially PCa [33]. Overwhelming evidence confirms the role of chronic inflammation in PCa aetiology, which includes potential stimuli for prostatic inflammation, inflammatory pathways and cytokines [34]. All these findings demonstrate that the active compounds in Chinese herbal medicines exert their therapeutic effects on PCa by modulating the biological processes of their protein targets.

Additionally, to find the intersection of DEGs in the prostate tumor and the target genes of the PCa-related herbs, we firstly identified 1880 DEGs in GSE134073 (Figure 4A) and then, a total of 20 overlapped genes between DEGs in the prostate tumor and the target genes of the PCa-related herbs were obtained (Figure 4B). In final, five hub genes including CCNA2, CDK2, CTH, DPP4 and SRC were screened, which are associated with human PCa prognosis. The expression analysis of these hub genes in PCa and normal tissues reveals that CCNA2, CDK2, CTH and DPP4 are unregulated in PCa tissue compared with normal tissue, while SRC is down-regulated in PCa tissue compared with normal tissue (Figure 5A–5F). In addition, the Kaplan-Meier survival analysis, immune relationship, immunohistochemistry, tumor and normal sample expression results also further verified these hub genes (Figure 5G–5K), demonstrating that these hub genes may become a biomarker and therapeutic target for accurate diagnosis and treatment of PCa in the future.

**Figure 10:**
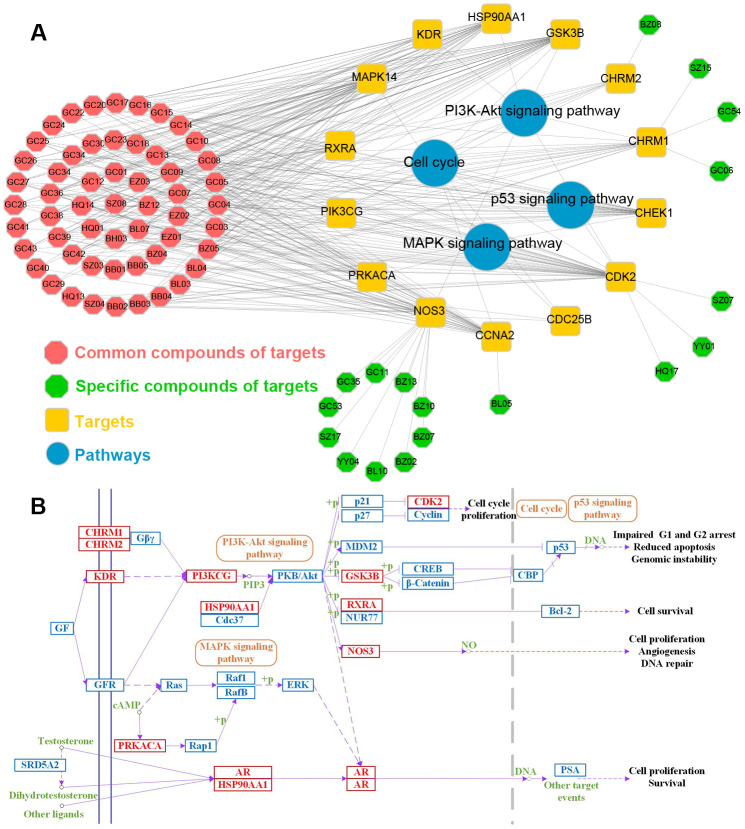
(**A**) The Compounds pathway network. The target node and the pathway node are linked. The yellow rectangle, red circle and octagon represent the target, pathway and compound respectively. (**B**) Distribution of ten herbs targets in compressed PCa pathway.

Besides, to further explore the major signaling pathways implicated in treatment of PCa by ten herbals, 4 PCa-related biological pathways were extracted from KEGG analysis. Using the targets and their corresponding pathways, the C-T-P network was established (Figure 10A). The yellow rectangles, blue circles and octagons represent targets, pathways and compounds, respectively. As shown in Figure 10, major pathways are regulated by multiple target proteins, many of which have been reported as appropriate target pathways for PCa therapies, such as PI3K-Akt, Cell cycle, MAPK and p53 signaling pathways. Moreover, in order to verify the systemic effects of these ten herbs on diseases, a comprehensive “PCa-related pathway” was assembled based on the current understanding of the pathology of PCa diseases (Figure 10B).

As displayed in Figure 10A, of the 14 targets, 9 were involved in PI3K-Akt signaling pathway, indicating that PI3K-Akt plays a key role in PCa therapy. Accumulating stuides reveal that PI3K-Akt pathway can be activated not only by the genetic mutation and amplification of key pathway components, but also by receptor tyrosine kinases [53]. Based on its role as a key regulator of cell survival, apoptosis, cell proliferation and immune activation, PI3K-Akt signaling pathway has become a central factor in the growth and progression of specific malignant tumors. Cell cycle, the pathway by which cells develop and proliferate, lies at the heart of cancer, such as PCa. In cancer, as a result of genetic mutations, apoptotic process malfunctions, resulting in uncontrollable cell proliferation and abnormal Cell cycle. Different cyclins may activate multiple kinases, including cyclin-dependent kinases (CDKs), and particular CDK complexes are required for passage out of a specific phase of Cell cycle [54]. The MAPK and p53 signaling pathways also play critical roles in tumor progression and PCa cell growth [55, 56].

All in all, these findings reveal the importance of PI3K-Akt, cell cycle, MAPK and p53 signaling pathways in PCa induction. Because these four pathways have a variety of functions, such as cell growth, cell proliferation, apoptosis regulation and immune activation, we speculate that these ten herbs may disrupt these pathways, thus showing direct anticancer therapy, immunotherapy and anti-inflammatory strategies. Candidate compounds may mediate the interaction and crosstalk between different pathways through target proteins, indicating that ten herbs may play synergistic roles in different pathways [7]. At last, several targets belong to more than one signaling pathway, indicating that they can interact with multiple pathways regulating the progress of PCa.

## CONCLUSIONS

Presently, a comprehensive approach combining bibliometric analysis, pharmacokinetic screening, target fishing, network and bioinformatics analysis was employed to elucidate the mechanisms of the anti-PCa related herbal medicines. The predicted results were also computationally validated through molecular docking as well as MD simulations. The main findings of this paper are listed in the following.

1. A total of 109 potential ingredients and 139 corresponding targets are obtained from 10 anti-PCa related herbal medicine through bibliometric analysis, ADME screening and target fishing.
2. GO and T-F network analysis show that the biological processes of these targets are mainly associated with the inflammation response, modulating the immune system and direct anti-cancer effects, suggesting that these 10 herbs have therapeutic effects on PCa from these three aspects.
3. Bioinformatics analysis reveal five hub genes including CCNA2, CDK2, CTH, DPP4 and SRC, which are validated by the MD simulations, are expected to be potential prognostic markers to improve PCa survival and prognostic accuracy.
4. The C-T-P network analysis of anti-PCa herbs showed that herbal medicines may simultaneously target PI3K-Akt, MAPK, p53 and cell cycle signaling pathways to achieve the effect of treating diseases, providing useful clues to make more effective therapeutics against PCa.

To sum up, this work offers an integrative analysis to study the herbal drugs and understand the action mechanism of herbal medicines for treating PCa from molecular level to pathway level, which provides new ideas for exploring new drug treatments for complex diseases.

## Supplementary Material

Supplementary Table 1
